# A Registered Dentist Sued

**Published:** 1899-02

**Authors:** 


A Registered Dentist named Frederick Mastin, carrying on business under the style of the Melbourne Dental Company, was recently sued in the County Court for £99 damages, under the following circumstances: Plaintiff stated that, on April 26th last, he called on defendant to have a tooth extracted, and, before submitting to the operation, asked what preparation was intended to be used to deaden the pain. Defendant said he used a preparation of his own, the component parts of which he would not disclose for £500, and assured him that it would have no injurious after effects. Plaintiff then consentented to have the tooth extracted. Next morning his tongue was very much swollen, and three days later the gums commenced to bleed. A medical man plugged the cavity in the gum, but it became worse, and he was unable to do anything for six weeks. Medical evidence for the plaintiff was directed to show that the injury might have been due to impurity in the preparation, or to the use of a syringe that had not been properly sterilized. For the defense one medical man asserted that the symptoms experienced might have followed the extraction of a tooth where no solution had been used, and another that the symptoms might have arisen from the state of the gums. Judgment was eventuslly given for defendant with costs.—*Pharmaceutical Journal.*

